# Infectious episodes during pregnancy, at particular mucosal sites, increase specific IgA1 or IgA2 subtype levels in human colostrum

**DOI:** 10.1186/s40748-019-0104-x

**Published:** 2019-06-11

**Authors:** Erick Sánchez-Salguero, Geovanni Kaleb Mondragón-Ramírez, Julio C. Alcántara-Montiel, Arturo Cérbulo-Vázquez, Xóchitl Villegas-Domínguez, Víctor Manuel Contreras-Vargas, María del Rocío Thompson-Bonilla, Héctor Romero-Ramírez, Leopoldo Santos-Argumedo

**Affiliations:** 1 Department of Molecular Biomedicine, Center for Research and Advanced Studies (CINVESTAV), National Polytechnic Institute (IPN), Mexico City, Mexico; 2 Interdisciplinary Center for Health Sciences, Milpa Alta Unit (CICSUMA), National Polytechnic Institute (IPN), Mexico City, Mexico; 3 School of Higher Studies Zaragoza, National Autonomous University of Mexico (UNAM), Regional Hospital of High Specialty of Ixtapaluca (HRAEI), Mexico City, Mexico; 4 Faculty of Medicine, Plan of Combined Studies in Medicine (PECEM), National Autonomous University of Mexico (UNAM), Mexico City, Mexico; 5 Women’s Hospital, Ministry of Health (SSA), Mexico City, Mexico; 6 Departments of Gynecology and Genomic Medicine, Regional Hospital 1° de Octubre, Institute of Security and Social Services of State Workers (ISSSTE), Mexico City, Mexico

**Keywords:** Immunoglobulin A, IgA1, IgA2, Respiratory tract, Gastrointestinal tract, Colostrum, Maternal transfer

## Abstract

**Background:**

Colostrum is the primary source of maternal immunoglobulin A (IgA) for the newborn. IgA participates in protection and regulation mechanisms of the immune response at the neonate’s mucosa. Several studies have evaluated infectious diseases and vaccine protocols effects during pregnancy on maternal milk IgA levels, with the aim to understand lactation protecting effect on newborn. However, most of their results demonstrated that there were no differences in the total IgA levels. In humans, IgA has two subclasses (IgA1 and IgA2), they have an anatomical distribution among mucosal compartments, their levels vary after antigen stimulation and are also seen to describe differential affinities in colostrum. Although there are differences between IgA subclasses in several compartments, these studies have excluded specific colostrum IgA1 and IgA2 determination.

**Methods:**

We analyzed data from 900 women in Mexico City. With Pearson correlation, we compared the number of infectious episodes during their pregnancy that was associated with mucosal compartments (skin, respiratory and gastrointestinal tracts) and colostrum IgA subclasses.

**Results:**

We show a correlation between increased colostrum IgA1 levels and the number of infectious episodes at respiratory tract and the skin. In contrast, infections at the gastrointestinal tract correlated with increased IgA2 amounts.

**Conclusions:**

**I**nfections present during pregnancy at certain mucosal site increase specific IgA subclasses levels in human colostrum. These results will help in understanding infections and immunizations effects on maternal IgA at the mammary gland, and their impact on the development and protection of the newborn.

**Electronic supplementary material:**

The online version of this article (10.1186/s40748-019-0104-x) contains supplementary material, which is available to authorized users.

## Background

At birth, humans are exposed to the most important and diverse antigenic contact during our lives. Newborns interact with diverse array of microorganisms and environment molecules and learn to distinguish between commensal microbiota and potential pathogens. This interaction is seen to drive a susceptibility window for acute infections [1].

Through colostrum, newborns receive the highest amounts of defense, stimulation, and development molecules, during the first three days postpartum from their mothers. These factors include immunoglobulins, cytokines, chemokines, growth factors, etc. [2, 3]. Among these molecules, colostrum is considered as the primary source of immunoglobulin (Ig) A [4, 5]. Through lactation, IgA in the serum is seen to be associated with positive newborn health benefits, that includes protection against microorganisms [6], microbiota selection [7] and reduced risks of chronic, inflammatory and allergic diseases [8, 9].

Maternal immunization during pregnancy is seen to induce antibodies production in serum and secretions (e.g., milk) [10–12]. Several controlled clinical studies have evaluated the effects of vaccine protocols and infectious diseases during pregnancy on colostrum and milk IgA [13, 14]. Most of their results have determined that colostrum and milk IgA levels are independent of infectious episodes numbers during pregnancy and, in some cases, also have provided contradictory information [15–18].

In humans, IgA is present in two subclasses (IgA1 and IgA2) and these IgA’s are differentially distributed at different mucosal tracts. Their secretions are also seen to vary according to where the plasma cells were recruited during the chemokines and integrins expression in these tissues [19]. IgA1 is predominantly found in the upper respiratory tract, skin, serum, and saliva; in contrast, IgA2 is more abundant in the small and large intestine. Post antigen stimulation, there is an increase of IgA subclasses at the distal mucosal compartments, and their secretions are found to vary based on their induction site [20]. Ladjeva et al. [21] in their research described significant individual variations in IgA1 and IgA2 subclasses distribution in 18 colostrum samples. They also analyzed IgA reactivity against polysaccharides and proteins. Both IgA1 and IgA2 were reactive against lipopolysaccharide, but specificity for proteins was found within IgA1. However, few other studies have also analyzed the infectious episodes effect on colostrum IgA subclasses levels.

Therefore, our study mainly aimed to evaluate the effects of infections during pregnancy on colostrum IgA subclasses levels. The quantitative enzyme-linked immunosorbent assay (ELISA) technique was used to measure colostrum Ig isotypes from 900 women in Mexico City, with an emphasis on IgA1 and IgA2 levels and their association with infectious episodes (IE) numbers at mucosal tracts.

## Methods

### Calculation of population size (n)

The population size (n) value was calculated considering the number of births during sampling lapse in Mexico City in according with Instituto Nacional de Estadística, Geografía e Informática (INEGI), 95% confidence interval (Z = 1.96), 5% error and also bearing in mind the infectious diseases incidence in pregnant women population.

### Population selection

The present research was an analytical, cross-sectional and correlational study. This study included 900 pregnant, clinically healthy women set (Table 1) who delivered in one of the three different hospitals of Mexico City: Hospital Regional 1° de Octubre ISSSTE (HR 1° Oct, 21%), Hospital Regional de Alta Especialidad de Ixtapaluca (HRAEI, 40%) or Hospital de la Mujer (HMuj, 38.77%). All procedures were explained to women, relatives or husbands, who signed a consent form, that followed the revised Helsinki Declaration (2013) [22]. Clinical information related to infectious episodes in skin, respiratory, gastrointestinal or urogenital tracts during pregnancy was obtained from each patient by a personal interview and from their clinical study evaluations. Information depicted in the individual clinical reports were validated with the data obtained from their interview during the sampling stage.

**Table 1:** Population study characteristics

Characteristic	Frequencies (percentages)
Age of mothers	Mean = 24.96 ± 6.613
Geographical origin	Mexico City = 873 (97%)
	Other = 27 (3%)
Delivery method	VD = 456 (50.67%)
	C-section = 444 (49.33%)
Number of parturitions	1st = 399 (44.33%)
	2nd = 287 (31.89%)
	3rd = 146 (16.22%)
	4th = 68 (7.56%)
Hospital	HR 1° Oct = 193 (21.44%)
	HRAEI = 359 (39.89%)
	HMuj = 349 (38.77%)
Epidemiological group	H group = 423 (47.00%)
	IE group = 477 (53.00%)

### Inclusion and exclusion criteria

This study included women between 14 and 46 years old, a full-term pregnancy (38–42 weeks), vaginal delivery (VD) or cesarean section (C-section) and single product parturition. Exclusion criteria included the presence of maternal chronic disorder, previous abortions, preterm pregnancy, multiparous parturition, women under hormonal treatment or acute disease diagnosis three weeks prior to the delivery.

### Sample collection

All samples were collected between July 2016 and August 2017 from mothers during the first 72 h postpartum. Two milliliters of colostrum were obtained from each patient using sterile tubes (Round-Bottom Polypropylene Tubes with Caps, Stemcell Technologies© Vancouver, Canada) and were transported on ice to the laboratory. Later these samples were centrifuged at 350 g for 10 min at 4 °C (Allegra™ X-22R Benchtop Centrifuges Beckman Coulter Life Sciences, Indianapolis, IN, USA) and the aqueous phase was separated from the lipid phase and its cellular components. Then the aqueous phase was re-centrifuged at 2000 g for 30 min at 4 °C and were frozen, stored at − 70 °C until further use.

### Quantification of total immunoglobulins (total Ig)

The ELISA was used to quantify colostrum IgA, IgA1, IgA2, IgM and IgG concentrations. Ninety-six-well plates (Immuno Plate MaxiSorp, Thermo Scientific, NY, USA, Cat. Num. 1132249) were coated with 100 μL of capture monoclonal antibody (mouse anti-human Ig light chain, Abcam®, Cambridge, UK, Cat. Num. ab1942) in 1 μg/mL phosphate buffered saline (PBS) and were incubated overnight at 4 °C. Between each reaction step, the plates were washed with 200 μL of 0.5% polyoxyethylene sorbitan monolaurate (Tween 20, Sigma-Aldrich Company®, USA, Cat. Num. 9005-64-5) in PBS (PBS-T) for eight times. The nonspecific-binding free sites were blocked with 1% bovine serum albumin (BSA, Sigma-Aldrich Company®, USA, Cat. Num. 9048-46-8) diluted in PBS-T (200 μL per well) by incubation during 30 min at 37 °C. Samples were processed by 10-fold serial dilutions starting at ratios of 1:100, 1:1000 and 1:10,000. For standard curves 100 *μ*L of IgA1 (human protein IgA1, Abcam®, Cat. Num. ab91020), IgA2 (human protein IgA2, Abcam®, Cat. Num. ab91021), IgM (human protein IgM, Abcam®, Cat. Num. ab91117) or IgG (natural human IgG, Abcam®, Cat. Num. ab91102) standard solutions were added and incubated for 2 h at 37 °C. For specific Ig isotypes detection, 100 μL per well of biotinylated antibodies anti-IgA (rabbit polyclonal antibody, Abcam®, Cat. Num. ab97218), anti-IgA1 (monoclonal mouse antibody, Abcam®, Cat. Num. ab99796; 1:2500), anti-IgA2 (monoclonal mouse antibody, Abcam®, Cat. Num. ab128731; 1:2000), anti-IgM (monoclonal mouse antibody, Abcam® Cat. Num. ab49655) and anti-IgG (goat anti-human, Invitrogen®, Massachusetts, MA, USA, Cat. Num. 81–245,062) were added and incubated in the same experimental conditions. Later 100 μL streptavidin-horseradish peroxidase (HRP) complex (Streptavidin HRP, Abcam®, Cat. Num. ab7403) dilution was added and incubated for 1 h at 37 °C. The presence of labeled antibodies was revealed using 100 *μ*L per well of chromogenic substrate 3′3″-5–5-tetramethylbenzidine (TMB ELISA substrate, Abcam®, Cat. Num. 171523) and the reaction was terminated by adding 100 μL of 0.2 M sulfuric acid per well. Post this the absorbance was measured in a spectrophotometer Microplate ELISA plate reader (Sunrise, Tecan® TX, USA) at 450 nm. IgA, IgA1, IgA2, IgM or IgG standard curves were obtained for each plate to determine each sample concentration by absorbance interpolation and dilution factor. The results were finally expressed in total Ig isotype milligrams per colostrum milliliter (mg/mL).

### Quantification of secretory immunoglobulins (SC-Ig)

The ELISA was also used to quantify total SC-IgA concentrations in colostrum. Ninety-six-well plates were coated with 100 μL of anti-human IgA (monoclonal antibody, Abcam®, Cat. Num. ab7400) in PBS and were incubated overnight at 4 °C. Washings, blocking and experimental samples addition are like the procedure described above. For the standard curve, purified human colostrum IgA (Natural human protein IgA from colostrum, Sigma-Aldrich® MO, USA, Cat. Num. I1010) was used. For specific SC-Ig detection, 100 *μ*L of biotinylated antibody anti-SC (Goat polyclonal antibody, Lifespan Biosciences®, Washington, WA, USA, Cat. Num. LS-C185086; 1:8000) in PBS was added per well and were incubated for an hour at 37 °C. The results were expressed in mg of SC-Ig per milliliter of colostrum.

### Statistical analysis

Correlation between infections numbers during pregnancy and colostrum Ig levels were determined by Pearson rank correlation coefficient. Mean values and 95% confidence intervals were calculated for each group data. Ig concentration distribution was analyzed by Kolmogorov-Smirnov test. Comparison of Ig concentrations among samples was performed by non-parametric Mann Whitney U test for two grouped data or Kruskal-Wallis test for multiple grouped data. All statistical test and graphics were developed using statistical program GraphPad Prism (GraphPad® Software version 7, La Jolla, CA, USA).

## Results

### Population data

Nine hundred healthy women, with a mean age of 24 years at parturition, were included in this study. Fifty percent of women had delivered their newborn by VD and the rest by C-section (49.33%). Forty-four percent of women had their first child, second one (31.89%), third one (16.22%) and the rest (7.56%) were considered multiparous women. Forty-seven percent of total population were women without registered infectious episodes during pregnancy (H group) and 53% of the total population were women with at least one infectious episode associated with respiratory, gastrointestinal or urinary tracts or skin during pregnancy (IE group; Table 1).

### IgA subclasses levels in human colostrum

The human colostrum IgA subclasses levels in Mexican population was measured by comparing the different IgA classes (IgA, SC-IgA, IgA1, and IgA2) against the other Ig’s (IgG and IgM; Additional file 1: Figure S1). None of colostrum Ig levels were seen to present normal distributions. Using the results of the previous researches [23, 24], we determined that IgA is the main isotype in colostrum. This study reported that IgA2 (mean = 11.87 ± 2.904 mg/mL) concentration in the colostrum was slightly higher than IgA1 (mean = 9.893 ± 1.577 mg/mL). In comparison with IgA, IgG and IgM concentrations were reported to be lower (IgG = 1.509 ± 0.8227 and IgM = 2.643 ± 0.761; Additional file 1: Figure S1 **(a)**). The total IgA levels in the colostrum (IgA mean = 24.76 ± 3.472 mg/mL) aided in concluding that 47.61% corresponded to the IgA2 and 39.95% to IgA1 (Additional file 1: Figure S1((**b**)). Most of human colostrum IgA (90.75%) was seen to be associated with SC (SC-IgA mean = 22.47 ± 3.15 mg/mL).

### IgA subclass levels did not show variations among population descriptors

Many descriptors played a pivotal role in identifying the characteristics of the population analyzed. The following descriptors were included:

- The hospital from where the mothers were recruited (HR 1°, HRAEI and HMuj; Additional file 2: Figure S2(**a**)).
- Delivery method: To check if physiological changes between delivery methods could affect the results (Additional file 2: Figure S2(**b**));
- Postpartum period: This variable was also evaluated as the protein functioning was seen to decrease in the colostrum with time (Additional file 2: Figure S2(**c**));
- Mothers age and parturition number: Age and parturition number (1st or uniparous, 2nd, 3rd, 4th child or multiparous) were also evaluated to check if the outcomes were altered with age or if multiparous women recorded different Ig levels than uniparous women (Additional file 1: Figure S2(**d,e**)).

The IgA subclasses levels comparison data did not show any statistical differences between groups. Hence with the descriptor outcomes, we concluded that Ig levels were independent of the descriptors that were used to stratify the study population.

### Colostrum IgA subclass levels vary in women with IE during their pregnancy

**Fig. 1:**
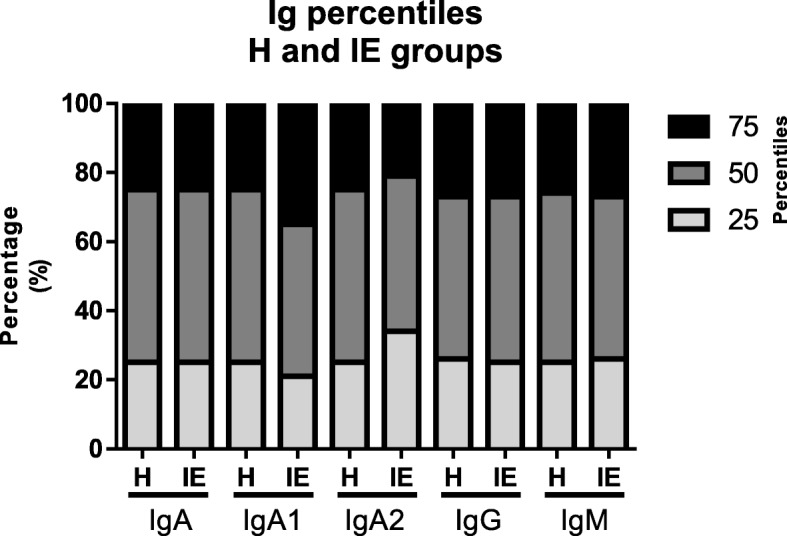
Ig levels distribution in the presence of IE. The bar graph shows the comparison among immunoglobulins data at 25 (light gray), 50 (gray) and 75 percentile (black) between H _group_ (*n* = 423) and IE _group_ (*n* = 477)

Previous studies reported that there were no statistical differences among total IgA in colostrum that correlated with infectious episodes presence during pregnancy. However, IgA subclasses quantification and its relation to IE during pregnancy have still not been reported. Hence later, the colostrum IgA subclasses quantities were compared between the H and IE groups. Results showed that IgA, IgG and IgM levels did not reveal any differences between groups. However, IE group seem to show a variation in colostrum IgA1 and IgA2 concentrations when compared with the women of the H group (Fig. 1).

Data stratification of the characteristics between H and IE groups demonstrated that there was no difference in the Ig levels between both the groups (Additional file 3: Figure S3). Hence from this point, the analysis was restricted to only evaluate Ig concentrations in colostrum between the two groups.

### Infectious episodes at specific mucosal tract increase colostrum IgA1 or IgA2 concentrations

**Fig. 2:**
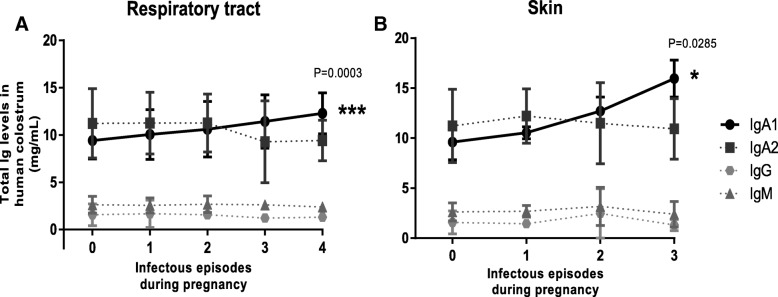
Colostrum Ig levels associated with IE in respiratory tract and skin. Graphics show colostrum Ig amounts from H (zero) or IE _groups_ (1, 2, 3 or 4 number of infectious episodes, respectively), associated with (**a**) respiratory tract (*n*_zero_ = 423, *n*_1_ = 142, *n*_2_ = 41, n_3_ = 23 and *n*_4_ = 10) and (**b**) skin (*n*_zero_ = 423, *n*_1_ = 25, *n*_2_ = 13 and *n*_3_ = 11). All values are represented as mean ± standard deviation (SD). All data are expressed in milligrams of Ig per milliliter of colostrum (mg / mL). Statistical analysis was performed using Pearson correlation test. *p < 0.05. ***p < 0.001

**Fig. 3:**
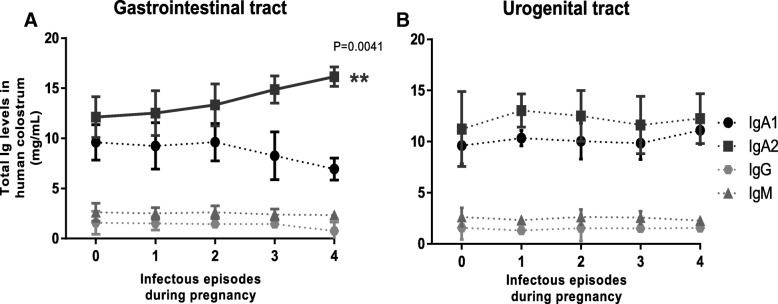
Colostrum Ig levels associated with IE in gastrointestinal and urogenital tracts. Graphics show colostrum Ig amounts with number of IE, associated with (**a**) gastrointestinal (*n*_zero_ = 423, *n*_1_ = 55, *n*_2_ = 18, *n*_3_ = 7 and *n*_4_ = 2) and (**b**) urogenital tract (*n*_zero_ = 423, *n*_1_ = 205, *n*_2_ = 56, *n*_3_ = 30 and *n*_4_ = 10). All values are represented as mean ± SD. All data are expressed in milligrams of Ig per milliliter of colostrum (mg / mL). Statistical analysis was performed using Pearson correlation test. **p < 0.01

We next determined the colostrum IgA subclasses concentration differences in the function of infectious episodes numbers associated in skin, mucosal compartments and urogenital tract. The results denoted that women who had infectious episodes in the respiratory tract had higher levels of colostrum IgA1 (H _group_ = 9.851 ± 1.569 mg/mL, IE_1_ = 9.925 ± 1.623 mg/mL, IE_2_ = 11.87 ± 2.971 mg/mL, IE_3_ = 10.43 ± 2.09 mg/mL and IE_4 or more_ = 12.28 ± 2.174 mg/mL; Fig. 2(a)). Similar behavior was also recorded when the infectious episodes were also associated with skin (H _group_ = 9.851 ± 1.569 mg/mL, IE_1_ = 10.54 ± 0.6067 mg/mL, IE_2_ = 12.71 ± 1.405 mg/mL and IE_3 or more_ = 15.95 ± 1.864 mg/mL; Fig. 2(b)). In sharp contrast, IgA2 concentrations increased with infections numbers associated with gastrointestinal tract (H _group_ = 9.851 ± 1.569 mg/mL, IE_1_ = 11.69 ± 3.145 mg/mL, IE_2_ = 12.84 ± 2.594 mg/mL, IE_3_ = 13.43 ± 2.256 mg/mLIE_4_ = 14.87 ± 1.359 mg/mL and IE_5 or more_ five or more IE = 16.16 ± 0.9687 mg/mL; Fig. 3(a)). However, in the cases of infectious episodes associated with the urogenital tract, concentrations of both IgA1 and IgA2 never showed any variations when compared with the H group (Fig. 3(b)).

## Discussion

According to this study results, we found that the number of infectious episodes during pregnancy affected specific IgA subclass concentrations. However, the possible mechanisms involved to explain these differences was not studied. A mechanism to explain this study results may involve IgA subclasses precursor cells (IgA+ PC) migration from inductor sites to mammary glands during pregnancy.

Under hormonal regulation [25], progesterone allows chemokine (C-C motif) ligand 25 (CCL25) and CCL28 expression and this, in turn, is seen to increase the mucosal vascular addressin cell adhesion molecule 1 (MadCAM-1) production and its higher expression on mammary glands endothelium during the final trimester of pregnancy. Chemokine receptors and specific integrins, like integrin alpha-4/beta-7 (α4β7) expression, may allow the IgA2+ PC cells to migrate to the mammary acini and reside there until lactation [26, 27]. At the same time, the estrogens are seen to induce and increase the vascular cell adhesion protein 1 (VCAM-1) production in the mammary gland, which may enhance the production of IgA1+ PC cells, with the expression of the integrin α4β1.

IE occurrence during pregnancy would increase Ig amounts in colostrum, due to the accumulation effect for each infectious episode. In response to an infection, IgA1+ or IgA2+ PCs production and migration depend on induction site at mucosal compartments [28, 29]. Respiratory tract and skin are sites where IgA1 production is higher than IgA2 [30]; meanwhile, IgA2 is the subclass in higher proportion along gastrointestinal tract [31].

Genitourinary tract is considered as an effector site of the immune response, without antibody-secreting cells significant recirculation to distal tissues. Antigen stimulations in this mucosa do not allow recirculation of secreting cells to other effector sites. Specific IgA subclass secretory cells arrival is favored only by an increase in the number of cells produced during infection. This characteristic may help to explain the absence of differences in colostrum IgA subclasses of women with IE in the genitourinary tract [32], although further research regarding this finding are necessary.

Statistical correlation does not necessarily mean a cause-consequence relation between data. The results only indicate that there is an intimate relation between mechanisms, since, biological processes are more complex and may involve more than two variable factors [33]. To demonstrate cause-consequence relation, more studies are necessary. As a clinical researching work, the current study had important limitations: it was based on infectious episodes incidence that mothers remembered and reported during their pregnancy, without considering others epidemiologic characteristics, like pregnancy time-lapse, duration and infection etiology. For this, a more systematic clinical study would be necessary to demonstrate a correlation between infections during pregnancy and its role in the maternal transference of specific IgA subclasses in mothers and babies during the first stages of life.

This research results provide pieces of evidence and additional information that contribute to solving controversial data about the effect of infections during pregnancy on Ig induction in mother and in her colostrum. This result is seen to help in establishing better programs of vaccination during pregnancy, delivery and/or after birth to induce and improve the protection of the mothers and newborns.

## Conclusion

The research outcomes aid in providing evidence to understand the effect of infections during pregnancy. Therefore, the same results can also suggest possible effects that specific IgA subclasses production would have on newborn protection. This research article provided experimental evidence in which episodes of infection effects in women during pregnancy has on the levels of IgA1 and IgA2 in colostrum.

## Additional files

Additional file 1: **Figure S1.** Quantification of the total Ig and SC-Ig present in colostrum. (a) The bar chart shows similar amounts of total IgA subclasses in colostrum, *n* = 900. (b) Comparative amounts of IgA types in colostrum (IgA, SC-IgA, IgA1 and IgA2), *n* = 900. Bars indicate mean ± SD. All data are expressed in milligrams of Ig per milliliter of colostrum (mg / mL). (PPTX 96 kb)

Additional file 2: **Figure S2.** Stratification of colostrum Ig levels in function of population descriptors. The bar chart shows comparative amounts of Ig in colostrum in function of (a) hospitals: HR 1° Oct (*n* = 175), HRAEI (*n* = 376) and HMuj (*n* = 349) and (b) delivery methods: VD (*n* = 457), C-section (*n* = 429) and non-specified (*n* = 14). Results are shown as mean ± SD. Statistical analysis was performed using the Mann-Whitney U test for non-parametric two independent data. Graphs points and lines shows comparison of Ig levels in function of (c) hours postpartum, since delivery moment (birth time is indicated with a black arrow) at 1–72 h postpartum; (d) Age of mothers range from 16 to 43 years old and (e) in function of number of parturition. Results are shown as mean ± SD. Statistical analysis was performed using Kruskal Wallis rank test for non-parametric > 2 independent data. No statistical difference was found in any case, hence multiple corresponding post hoc test was not performed. (PPTX 361 kb)

Additional file 3: **Figure S3.** Population descriptors between H and IE groups. The bar chart shows comparative amounts of Ig in colostrum between H (*n* = 423) and IE _groups_ (*n* = 477) groups. Hospitals: (a) H _group_ (HR 1° Oct (*n* = 25), HRAEI (*n* = 117) and HMuj (*n* = 281)) and (b) IE _group_ (HR 1° Oct (*n* = 168), HRAEI (*n* = 242) and HMuj (*n* = 68)). Delivery methods: (c) H _group_ (VD (*n* = 246), C-section (*n* = 170) and non-specified (*n* = 7) and (d) IE _group_ (VD (*n* = 211), C-section (*n* = 259) and non-specified (*n* = 7)). Results are shown as mean ± SD. Statistical analysis was performed using Mann-Whitney U test for non-parametric two independent data. Graphs points and lines display the comparison of Ig levels in colostrum between H (*n* = 423) and IE (*n* = 477) groups. Postpartum hours: (e) H group (birth time is indicated with a black arrow) at 1–72 h postpartum and (f) IE group (birth time is indicated with a black arrow) at 1-72 h postpartum. Parturition number: (g) H group and (h) IE group parturition episodes. Age of mothers: (i) H _group_ (16-43y) and (j) IE _group_ (16-43y). Results are presented as mean ± SD. Statistical analysis was performed using Kruskal Wallis rank test for non-parametric > 2 independent data. No statistical difference was observed in any case, hence multiple corresponding post hoc test was not conducted. (PPTX 595 kb)

## Abbreviations

C-section: cesarean section
ELISA: enzyme linked immunosorbent assay
IE: infectious episodes
IgA: immunoglobulin A
IgA1: immunoglobulin A subclass 1
IgA2: immunoglobulin A subclass 2
INEGI: Instituto de Estadística, Geografía e Informática
ISSSTE: Instituto de Seguridad y Servicios Sociales de los Trabajadores del Estado
VD: vaginal delivery
