# Supplementary figures and images for: Infectious episodes during pregnancy, at particular mucosal sites, increase specific IgA1 or IgA2 subtype levels in human colostrum
Source: Matern Health Neonatol Perinatol. 2019 Jun 11;5:9. doi: 10.1186/s40748-019-0104-x (PMC6558797; doi:10.1186/s40748-019-0104-x)

## Slide 1
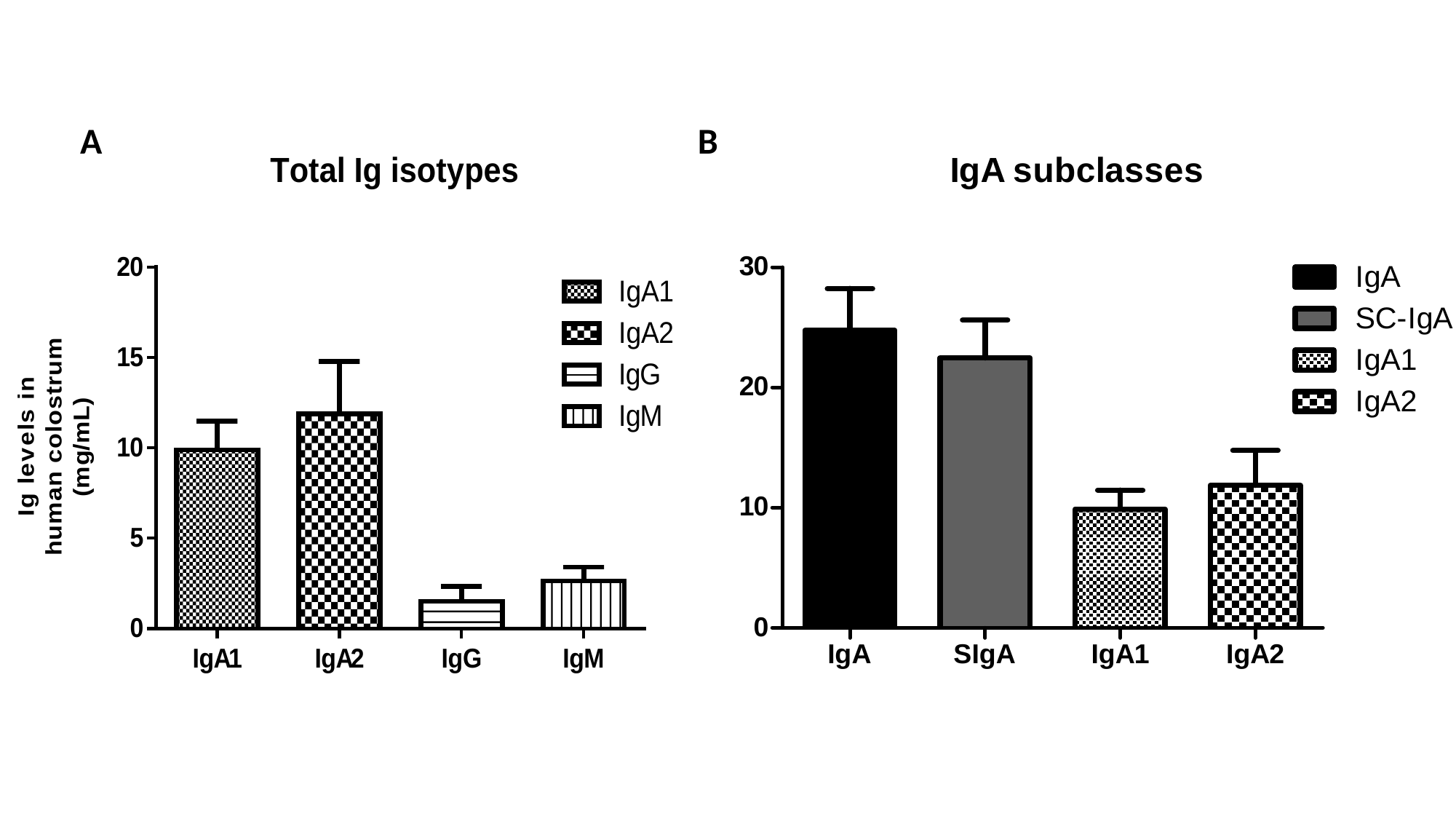

A
B

Supplement: Supplementary file 1 — Figure S1. Quantification of the total Ig and SC-Ig present in colostrum. (a) The bar chart shows similar amounts of total IgA subclasses in colostrum, n = 900. (b) Comparative amounts of IgA types in colostrum (IgA, SC-IgA, IgA1 and IgA2), n = 900. Bars indicate mean ± SD. All data are expressed in milligrams of Ig per milliliter of colostrum (mg / mL). (PPTX 96 kb) [file 40748_2019_104_MOESM1_ESM.pptx]

## Slide 1
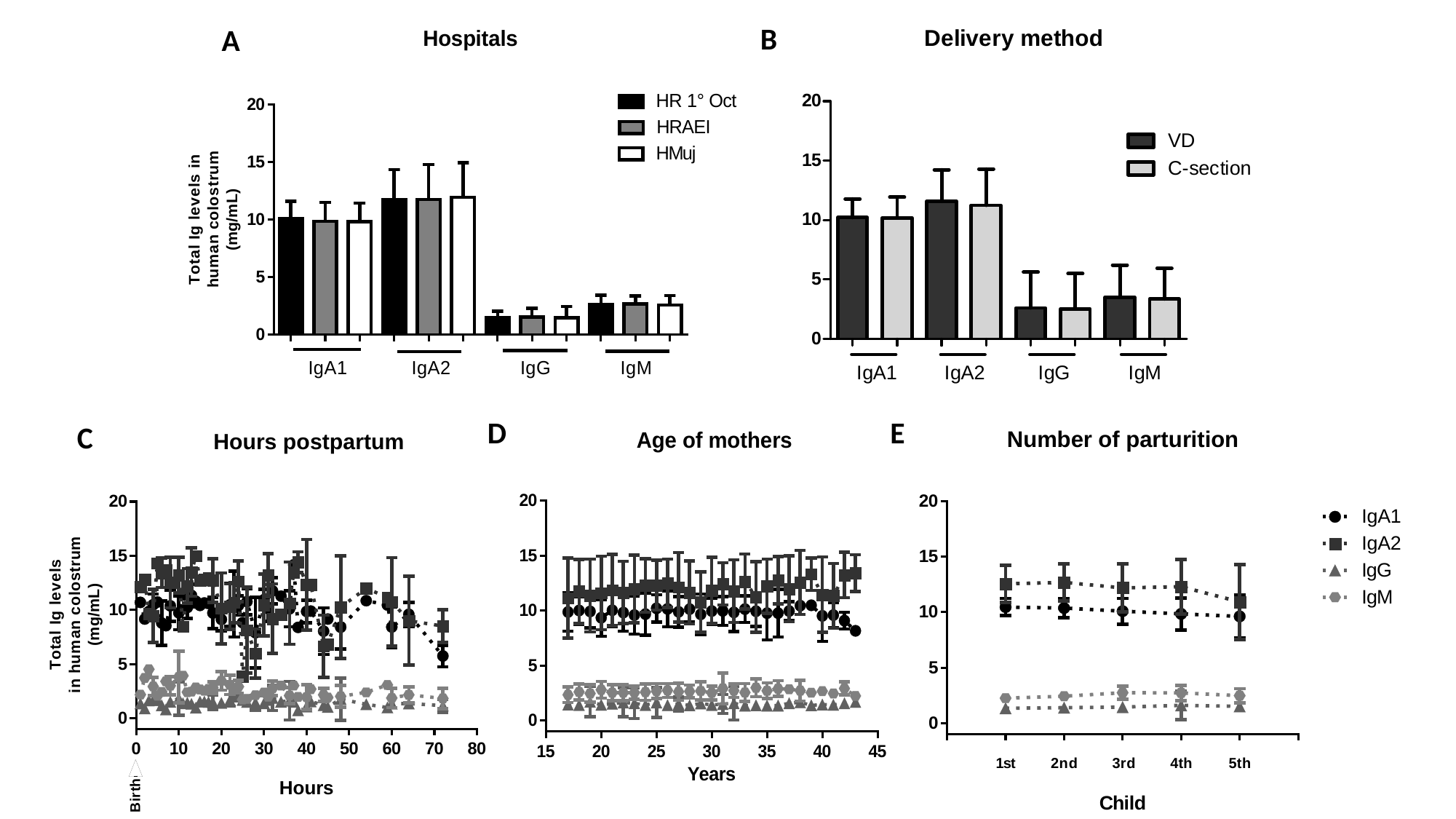

B
A
E
D
C

Supplement: Supplementary file 2 — Figure S2. Stratification of colostrum Ig levels in function of population descriptors. The bar chart shows comparative amounts of Ig in colostrum in function of (a) hospitals: HR 1° Oct (n = 175), HRAEI (n = 376) and HMuj (n = 349) and (b) delivery methods: VD (n = 457), C-section (n = 429) and non-specified (n = 14). Results are shown as mean ± SD. Statistical analysis was performed using the Mann-Whitney U test for non-parametric two independent data. Graphs points and lines shows comparison of Ig levels in function of (c) hours postpartum, since delivery moment (birth time is indicated with a black arrow) at 1–72 h postpartum; (d) Age of mothers range from 16 to 43 years old and (e) in function of number of parturition. Results are shown as mean ± SD. Statistical analysis was performed using Kruskal Wallis rank test for non-parametric > 2 independent data. No statistical difference was found in any case, hence multiple corresponding post hoc test was not performed. (PPTX 361 kb) [file 40748_2019_104_MOESM2_ESM.pptx]

## Slide 1
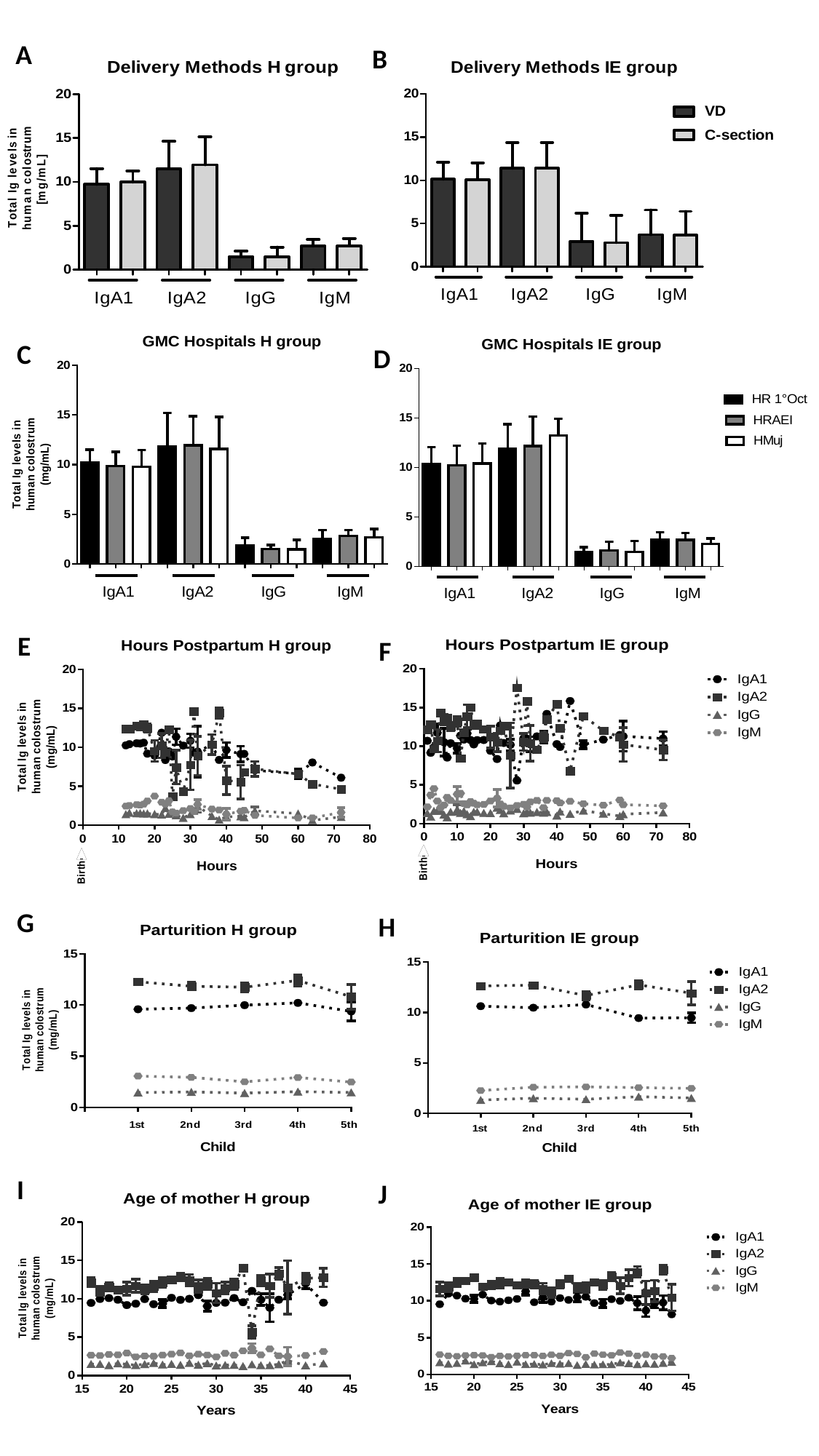

A
B
C
D
E
F
G
H
I
J

Supplement: Supplementary file 3 — Figure S3. Population descriptors between H and IE groups. The bar chart shows comparative amounts of Ig in colostrum between H (n = 423) and IE groups (n = 477) groups. Hospitals: (a) H group (HR 1° Oct (n = 25), HRAEI (n = 117) and HMuj (n = 281)) and (b) IE group (HR 1° Oct (n = 168), HRAEI (n = 242) and HMuj (n = 68)). Delivery methods: (c) H group (VD (n = 246), C-section (n = 170) and non-specified (n = 7) and (d) IE group (VD (n = 211), C-section (n = 259) and non-specified (n = 7)). Results are shown as mean ± SD. Statistical analysis was performed using Mann-Whitney U test for non-parametric two independent data. Graphs points and lines display the comparison of Ig levels in colostrum between H (n = 423) and IE (n = 477) groups. Postpartum hours: (e) H group (birth time is indicated with a black arrow) at 1–72 h postpartum and (f) IE group (birth time is indicated with a black arrow) at 1-72 h postpartum. Parturition number: (g) H group and (h) IE group parturition episodes. Age of mothers: (i) H group (16-43y) and (j) IE group (16-43y). Results are presented as mean ± SD. Statistical analysis was performed using Kruskal Wallis rank test for non-parametric > 2 independent data. No statistical difference was observed in any case, hence multiple corresponding post hoc test was not conducted. (PPTX 595 kb) [file 40748_2019_104_MOESM3_ESM.pptx]
